# Editorial: The Impact of Countries' Economy and Wealth on their Research Activities

**DOI:** 10.3389/frma.2022.1129373

**Published:** 2023-01-12

**Authors:** Stanley I. R. Okoduwa, Giuseppe Calignano, Manuel J. Cobo

**Affiliations:** ^1^Directorate of Research and Development, Nigeria Institute of Leather and Science Technology, Zaria, Nigeria; ^2^Department of Biochemistry, School of Basic Medical Sciences, Babcock University, Ilishan-Remo, Nigeria; ^3^Department of Organisation, Leadership and Management, Inland Norway University of Applied Sciences, Lillehammer, Norway; ^4^Department of Computer Science and Artificial Intelligence, University of Granada, Granada, Spain

**Keywords:** research activities, research funding, countries' economy, policymaker, collaborative research, research challenges, interdisciplinary research, barriers to research

Research activity encompasses the collation, analysis, and interpretation of information aimed at improving human knowledge and understanding. This is subject to the economy and wealth of academic and research institutions and, consequently, to the country to which they belong. In countries with <$1,035 per capita gross income (GNI), research activities suffer serious setbacks (Naciri, 2018). Such economic conditions often result in the scarcity or even absence of research funds. Insufficient research funding can also be interrelated with unsatisfactory educational outcomes, poor infrastructures, and unfavorable government policies at the national level (Okoduwa et al., 2018). Government policies can deter the implementation of quality research. For instance, in Nigeria, the amendment of section 20 of the “Education Trust Fund (ETF)” establishment Acts—which is currently named “Tertiary Education Trust Fund (TETFund)”—excludes research-focused institutions from benefiting from research funding [Federal Republic of Nigeria Official Gazette (FRNOG), 2011; Tertiary Education Trust Fund (Establishment, Etc.) (TETFund) Act, 2011]. Ultimately, the situation in which scholars conduct research can result in job dissatisfaction leading to the emigration of researchers in countries with more favorable conditions.

Emerging evidence in recent reports suggests, however, that cross-collaborative and interdisciplinary research may ease the challenges faced by researchers with limited or no access to research funds. Collaboration and networking between academic and industrial researchers could represent a potential avenue for overcoming the lack of funds and for establishing ties at various geographical scales (Wagner and Leydesdorff, 2005; Mathews and Hu, 2007; Giuliani and Rabellotti, 2012; Calignano and Quarta, 2014; Okoduwa, 2017). It needs to be highlighted, of course, how international funding organizations and evaluators may perceive institutions in developing and low-income countries as generally inferior This perception may have a negative impact on the ability showed by a given institution to acquire vital external research funds (evaluators might tend to privilege more “reputable” organizations) and join winning research consortia (see, e.g., Breschi and Cusmano, 2004; Calignano, 2022).

In the present Research Topic, some of these points were tackled by four papers adopting a qualitative or conceptual approach, in which special attention was devoted to the tension between wealth and research funding in an international context. The common attitudes, perceptions, and barriers to research activities among scholars from institutions with limited research funds and financial support were addressed by Igiri et al.. This qualitative explorative analysis examined academics affiliated with various research and tertiary education institutions located in the six geographical regions making up Nigeria. The study investigated the productivity and challenges that researchers face to meet their job schedule. In particular, it was observed that Nigerian policymakers provide little or no research funds, thus pushing scholars to use their salaries and stipends to conduct research, publish articles and attend conferences before they are promoted. This primarily depends on strong external pressure, which is expressed by the axiom “publish or perish” (Van Dalen and Henkens, 2012). The findings of this study back the argument that neglecting the importance of research has contributed to underdevelopment in the country. Consequently, the authors recommend the federal government to prioritize research activities and establish a functional special research trust fund to oversee research funding in Nigeria.

There is no doubt that research and technology organizations (RTOs) have played a key role in several stories of national industrial development. In this regard, Sheikheldin proposes a conceptual framework of RTOs as super intermediaries since they play multiple intermediary roles in the triple helix of innovation (government, research and industry), the overlap of industrial and research policy, and the research-industry frontiers. Tanzania was used as a case study to explore whether investments in RTOs may potentially enhance the country's industrial development. The framework proposed in the article helps to understand and advance the role of RTOs in industrial development, with specific regard to developing countries.

The challenge of how to restructure the funding system for basic research to reinvigorate the indigenous innovative capacity has recently been one of the major concerns for the Chinese government. Interestingly, Bai et al. propose a conceptual framework to analyze how China's central government funding system for basic research has evolved since 1985. The authors were able to identify problems and challenges that China is currently facing in this regard, while they also provided insights on how these difficulties have affected the ability to achieve ground-breaking scientific results in the country. Bai et al. offer an analytical framework to examine the central government funding by considering two different dimensions: i.e., the drivers of basic research and the funding recipients.

Last but not the least, this Research Topic also focuses on the need to consolidate and upscale research capacity to drive the expected economic development of a country, while ensuring researchers' satisfaction in their work activities. Chukwudi thoroughly examined this aspect in her study. More specifically, the author observed that molecular research holds great promises for improving lives and living. Unfortunately, the heavy start-up capital required is a major setback in a developing country such as Nigeria, where the majority of the population lives below the poverty line and research funding is extraordinarily low. This paper examines the progression and challenges of undertaking molecular research in Nigeria, and how Nigerians are tackling such issues, by outlining the numerous difficulties faced by indigenous scientists operating in the field. Moreover, possible strategies on how policymakers and funding bodies might effectively support molecular research were also discussed in the study.

In conclusion, the goal of this Research Topic is to stimulate knowledge production in response to discussions on attitudes, perceptions, and barriers to research activities among scholars facing funding challenges. The articles presented in this Research Topic have brought together knowledge shared among researchers working in settings where research funding is either limited or unavailable.

## Author contributions

SIRO got the concept and design of the study and wrote the manuscript draft. GC and MJC participated in executing the research. All authors listed have made a substantial, direct, and intellectual contribution to the work and approved it for publication.
